# The Current Progress of Psychiatric Genomics

**DOI:** 10.14789/jmj.JMJ21-0038-R

**Published:** 2022-02-16

**Authors:** MASAKI NISHIOKA

**Affiliations:** 1 Department of Psychiatry, Faculty of Medicine, Juntendo University, Tokyo, Japan; 1 Department of Psychiatry, Faculty of Medicine, Juntendo University, Tokyo, Japan; 2 Department of Molecular Pathology of Mood Disorders, Faculty of Medicine, Juntendo University, Tokyo, Japan; 2 Department of Molecular Pathology of Mood Disorders, Faculty of Medicine, Juntendo University, Tokyo, Japan; 3 Laboratory for Molecular Pathology of Psychiatric Disorders, RIKEN Center for Brain Science, Saitama, Japan; 3 Laboratory for Molecular Pathology of Psychiatric Disorders, RIKEN Center for Brain Science, Saitama, Japan

**Keywords:** genomics, psychiatric disorder, bipolar disorder, schizophrenia, autism spectrum disorder (ASD)

## Abstract

Psychiatric disorders such as bipolar disorder and schizophrenia are highly heritable. While the genetic contribution to psychiatric disorders is quite sure, specific genetic factors contributing to particular conditions have long been a mystery. Empowered by the initial report of the Human Genome Project, the analysis of the comprehensive set of the human genome, called “genomics,” became possible. Subsequent development of large-scale genomic technologies enabled us to elucidate various disease-related genetic information, accelerating our understanding of various diseases. Genomic research on psychiatric disorders is not an exception. In this Review, I introduce significant advancements in psychiatric genomics with a special focus on our investigation of bipolar disorder. International consortiums and advocacy groups accelerate psychiatric genomics, increasing the sample size and statistical power for robust findings. The genetic architecture of schizophrenia has been elucidated in both common and rare variant studies. The genetic architecture of autism spectrum disorder (ASD) has been elucidated mainly by rare variant analysis. As to bipolar disorder, common variant analysis precedes rare variant analysis, but we are struggling to elucidate relevant rare variants. While the genomic approach has explained specific genetic factors for particular disorders, overlapping risk genes or pleiotropy has been observed more than expected. The boundary in the current nosology of psychiatric disorders is more or less challenged. To understand the genotype-phenotype relation more deeply, an attempt to understand phenotypes based on genotypes, called the “genotype first” approach, has started. I discuss this new approach for better understanding and treatment of psychiatric disorders.

## Introduction: psychiatry and genetics

Psychiatric disorders such as bipolar disorder and schizophrenia are global medical problems afflicting many individuals with severe suffering. The societal cost of bipolar disorder and schizophrenia is estimated high among various medical diseases^1)^. Bipolar disorder afflicts the patients and their families with severe depression and problematic behaviors from manic episodes. Schizophrenia leads to unbearable suffering through annoying hallucinations, persecutory delusions, social withdrawal, and cognitive decline. Despite the patients’ woes, we have limited choice of therapeutic strategy. The adverse effects of the medications are also problematic. Besides, the effects of psychiatric medications are serendipitously discovered, not based on our biological understanding of the mechanisms of psychiatric disorders. We need to understand the biological mechanisms of psychiatric disorders to find a new therapeutic strategy and overcome the suffering of the patients due to psychiatric symptoms.

Psychiatric disorders such as bipolar disorder and schizophrenia are highly heritable^2)^. Observing that many patients have families or relatives with similar mental problems, clinicians have long noticed this fact. The high heritability of psychiatric disorders is confirmed by the diagnostic concordance rate of monozygotic twins. Monozygotic twins have the same germline genetic information. The high rate of diagnostic concordance means that the contribution of genetic factors is highly relevant to psychiatric disorders. For example, the diagnostic concordance of bipolar disorder is 40-50%^2)^, while the lifetime morbidity of bipolar disorder is around 1%. However, we have not fully understood the biological background of this high heritability. Specific genetic information and biological mechanisms to the onset of psychiatric disorders remain unknown.

Genetic information is coded as sequences of bases in DNA. All the creatures on Earth adopt this system. *Homo sapiens* is not an exception. After the first proposal of the double helix structure of DNA as media of genetic information, researchers have been long pursuing what specific features of DNA contribute to the phenotypes of the individual. In psychiatry, the phenotypes in the individuals correspond to the diagnosis of psychiatric disorders and their symptoms. Psychiatric researchers desire to know specific characters of DNA that contribute to psychiatric disorders. The initial effort of this endeavor started from single nucleotide polymorphisms (SNPs). SNP is one type of genetic variant as a conversion from one base to another with a frequency of one percent or more in the general population. This was a good start point because SNP is relatively easy to detect. However, the initial effort to hunt disease-related SNPs was not fruitful^3)^. This effort is like fishing in the Pacific Ocean, and fishing all over the Pacific Ocean was not realistic at that time. To explore all over the Pacific Ocean, we needed a different approach.

## The birth of genomic analysis

The Human Genome Project was started in 1990 by the government of the United States. This project aimed at the complete catalog of the human genome^4)^, which spans around three Giga base pairs as one haplotype. Empowered by the Human Genome Project and subsequent development of large-scale DNA sequencing technology such as SNP chip and next-generation sequencing^5)^, comprehensive human genome analysis became realistic. This new approach is called “genomics.” The word “genome” means a whole set of genes, consisting of “gene” and “-ome” (a suffix meaning totality). In contrast to fishing in one spot, this approach can be compared to broad image capture by artificial space satellites. Capturing the images all over the Pacific Ocean became possible. Researchers are now armed with genomic technologies to investigate the human genome, finding a lot of variants associated with medical diseases. As the cost of genomic technologies declines, the sample size of genomic research increases, and the conclusion from the analysis becomes robust. Psychiatric genomics is not an exception.

**Figure 1:**
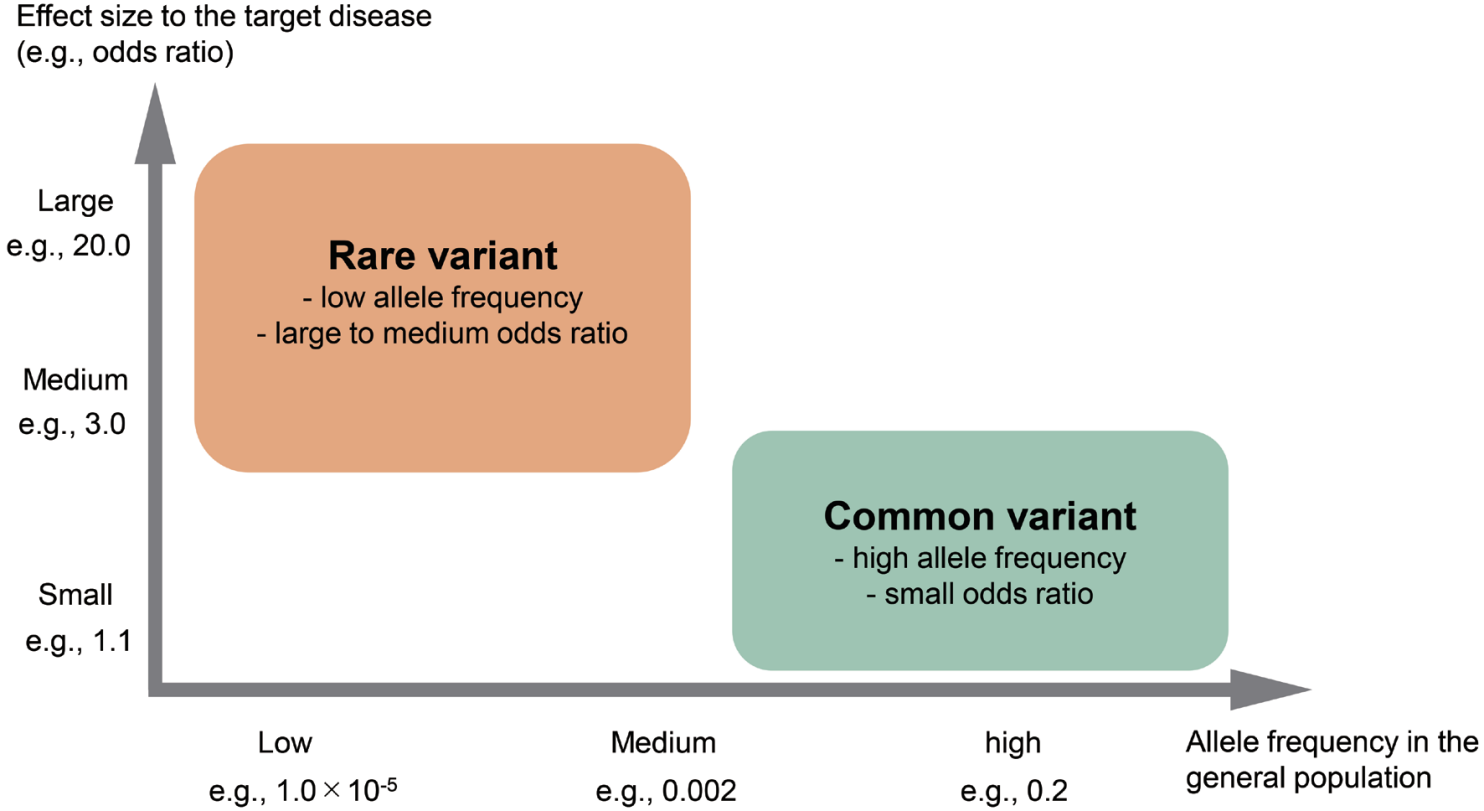
A schematic illustration of the disease-associated variants. The x-axis is the allele frequency in the general population, and the y-axis is the effect size to the target disease (e.g., odds ratio). Note that this illustration is a theoretical framework, and there are exceptions to this framework in reality.

A variant in the human genome receives different natural selection pressure with the phenotypes associated with it. Variants associated with severe diseases such as life-threatening pediatric cardiovascular diseases tend to be negatively selected, thus having a lower frequency in the general population. In contrast, variants scarcely associated with severe diseases are neutral and can have a high frequency in the general population by genetic drift. To put it the other way around, variants with higher frequency in the general population (common variants) tend to have minor effects on diseases; variants with lower frequency in the general population (rare variants) tend to have a more significant effect on diseases^6)^. Figure 1 illustrates the theoretical distribution of disease-associated variants related to their effect on the disease and their frequency in the general population. Note that this illustration is a theoretical framework, and there are some exceptions to this framework, such as *APOE* and Alzheimer's disease^7, 8)^. In general, the contribution of common variants is supposed to be more significant to common diseases (e.g., diabetes mellitus) than rare diseases (e.g., Mendelian diseases); the contribution of rare variants is supposed to be more significant to rare diseases than common diseases. Disease-associated common variants have been detected mainly by genome-wide association studies (GWAS) using SNP chips. SNP chips can genotype millions of SNPs simultaneously, while the investigation is limited to the pre-designed SNPs. Disease-associated rare variants have been detected mainly by whole-exome sequencing using massively parallel sequencing technology (so-called next-generation sequencing). Exome sequencing can detect variants in the exonic regions (protein-coding regions), including brand-new variants yet known before, but the cost of this technology is higher than SNP chips. The technical details of these technologies are themselves interesting, but they are beyond the scope of this Review. The technical details of such genomic technologies, including newer technologies, are reviewed elsewhere^9, 10)^.

The current psychiatric genomic investigation is advancing following this framework. We are now harvesting the fruit of the genomic analysis of psychiatric disorders to understand the biological mechanisms of psychiatric disorders^11, 12)^. Directly investigating the human brain is ethically and technically challenging. Biological insight from genetic studies complements this difficulty by providing indirect evidence for the etiology of psychiatric disorders. I review the major advancement of psychiatric genetics in the last decade, focusing on significant works on schizophrenia and autism spectrum disorder (ASD), and our investigation on bipolar disorder.

## Schizophrenia

Schizophrenia is a severe psychiatric disorder characterized by auditory hallucinations and persecutory delusions. Schizophrenia afflicts around one percent of the general population, afflicting the patients with painful experiences and social dysfunction due to cognitive decline. Schizophrenia is regarded as one of the major global medical issues because the societal cost of schizophrenia is high among other medical diseases^1)^. Schizophrenia has long been the most major disease concept in psychiatry and is also intensively investigated in genomic research. The prevalence of around one percent is on the threshold of common or rare diseases. Thus, genomic investigation of schizophrenia is advancing under two models: research to common variants as common diseases and rare variants as rare diseases.

Several hundred common variants associated with schizophrenia have been elucidated from the GWASs of international collaboration using SNP chips^13-16)^. Psychiatric Genomics Consortium (PGC) is the most influential international collaboration of psychiatric genomics^17)^, reporting several significant results^13-15)^. Precisely speaking, the associated common variants are not always the direct cause of the association but represent the genomic loci around them. We need to be cautious in interpreting the “associated genes” in these studies. The newest study of PGC (PGC3) consists of around 70,000 cases with schizophrenia and 240,000 controls, revealing 270 loci associated with schizophrenia^18)^. The effect size of each variant (or each genomic locus) is relatively small, with an odds ratio of 1.3 at most^18, 19)^, but the number of the association is biologically informative. The associated genes or loci are enriched in the gene sets related to synapse, especially those coding postsynaptic structure proteins. From the viewpoint of genetic studies of common variants, schizophrenia is a disease of synaptic dysfunction. Notably, the associated genes include *DRD2*, coding a subunit of dopaminergic receptors in the postsynaptic structure. One of the most promising hypotheses for the etiology of schizophrenia has been the dysregulation of the dopaminergic system^20)^. The studies of common variants support this long-held hypothesis in psychiatry.

Following the promising results of studies of common variants, schizophrenia-associated rare variants have been elucidated^21-28)^. The current understanding of rare variants for schizophrenia is mainly derived from studies using whole-exome sequencing. A meta-analytic effort from Broad institute aggregates an enormous volume of exome sequencing data of 24,000 cases and 97,000 controls from all over the world^29)^. Nine genes are robustly associated with schizophrenia through deleterious mutations in these genes: *SETD1A*, *CUL1*, *XPO7*, *TRIO* *CANCA1G*, *SP4*, *GRIA3*, *GRIN2A*, and *HERC1*. The deleterious mutations are mainly protein-truncating mutations such as nonsense or frameshift mutations. These mutations tend to be depleted or extremely rare in the general population, thus are ultra-rare variants. Notably, *SP4* and *GRIN2A* are deeply associated with schizophrenia through rare and common variants^18, 29)^. *GRIN2A* codes a subunit of NMDA receptors, one of the key components of glutamatergic neurotransmission. Another gene coding a key component of glutamatergic neurotransmission (AMPA receptor), *GRIA3*, is also robustly associated with schizophrenia through rare variant analysis^29)^. Dysfunction of glutamatergic neurotransmission is one of the most promising hypotheses for the etiology of schizophrenia^30, 31)^. The studies of both common and rare variants support this notion.

## Autism spectrum disorder (ASD)

ASD is a major neurodevelopmental disorder characterized by difficulties in social interaction/communication and restricted/repetitive behavior. This disease concept is highly spectrum, and the boundary between affected and unaffected status is difficult to define. Studies of rare variants mainly drive the genetics of ASD. While the number of ASD-associated common variants is few^32)^, the number of ASD-associated genes with rare variants is culminating. Exploration of rare variants in ASD is highly powered by studies on sporadic trios with ASD to investigate de novo mutations^33-40)^. De novo mutation is a newly arising mutation in the parents’ gametogenesis, detected as germline variants in the probands while not as germline variants in the parents. De novo mutation is a promising candidate to explain sporadic cases in which only the proband has the target disease (ASD), and the parents have no target disease.

Accumulating evidence has been elucidating the genes hit by de novo mutations (mainly protein- truncating mutations) robustly associated with ASD, including *CHD8*, *SCN2A*, and *ARID1B*, to name a few. The most extensive study of de novo mutations in ASD to date analyzed over 6,000 families with ASD and detected 102 genes as promising candidate genes for ASD^40)^. Simons Foundation Autism Research Initiative (SFARI) is aggregating and curating the genetic research results on ASD so far, listing several hundreds of candidate genes for ASD in a user-friendly website: https://gene.sfari.org/. Around 200 genes are listed as robustly associated with ASD in this curation. Advocacy groups such as SFARI are influential players as a funding agency and promoting agency for the general public in advancing genetic studies on ASD^41)^. Thanks to the effort of advocacy groups, rare variant studies on ASD are more advanced than studies on other disorders, exemplifying a research paradigm for studies on other disorders.

## Bipolar disorder

Bipolar disorder is a severe psychiatric disorder characterized by mood swings of depression and manic states. Bipolar disorder is relatively common, affecting around 1% of the population. Suffering from severe depression and problematic behavior from manic moods significantly impact patients’ social life. Elucidating the pathophysiology of bipolar disorder and the development of new treatments are required^42)^. Similar to the situation of schizophrenia, the prevalence of around 1% is on the threshold of common or rare diseases. Genomic investigation of bipolar disorder is also advancing under the two models: research to common variants as common diseases and rare variants as rare diseases.

Studies on common variants precede studies on rare variants in the genetics of bipolar disorder^43-46)^. GWASs on bipolar disorder have detected 64 loci associated with bipolar disorder using a consortium-based approach with aggregated 42,000 cases and 370,000 controls^44)^. As with the situation in schizophrenia, the effect size of each variant is relatively small. The odds ratio of the associated loci in bipolar is 1.15 at most, which is even smaller than schizophrenia. However, the number of the association is biologically informative, elucidating the gene sets related to synapse and ion channels. Synaptic structure and function are relevant to both bipolar disorder and schizophrenia. The phenotypes of bipolar disorder and schizophrenia are apparently different, but the correlation of associated common variants is notable between bipolar disorder and schizophrenia with a genetic correlation of around 0.68^44, 47^^-^^49)^. This high correlation is probably against most clinical psychiatrists’ intuition and should be the focus of future psychiatric genomics^50)^.

**Figure 2:**
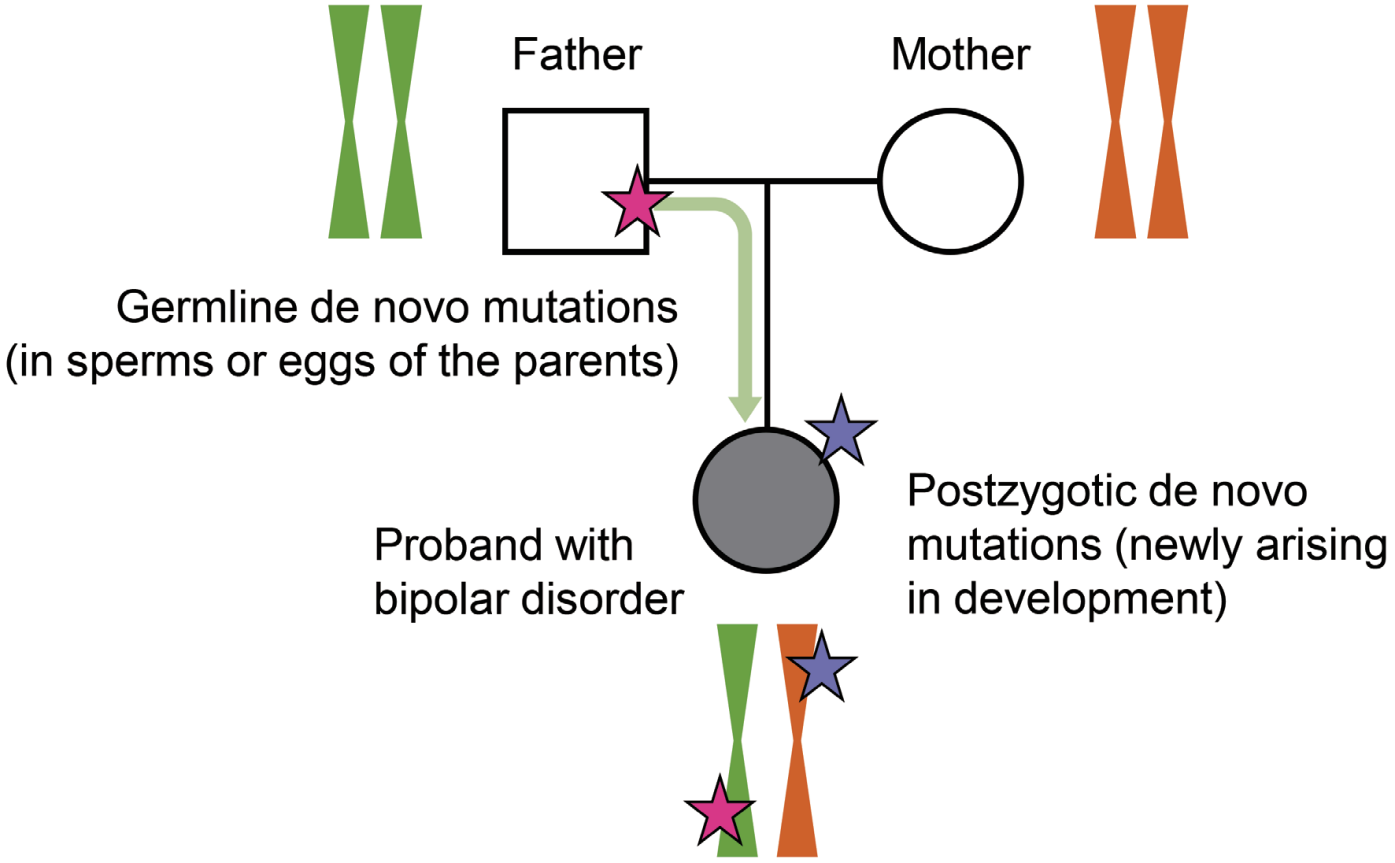
A schematic illustration of the two classes of de novo mutations: germline and postzygotic de novo mutations. The germline de novo mutations arise in the gametogenesis of the parents. The postzygotic de novo mutations arise in the developmental process of the proband after the fertilization.

In contrast to studies on common variants, studies on rare variants in bipolar disorder lags behind in sample size despite several pioneering studies^51-55)^. Therefore, we are investigating rare variants, especially de novo mutations, to elucidate the genetic architecture of bipolar disorder^53, 56)^. De novo mutations are subject to little natural selection and are thought to contain disease-associated mutations with significant effects. In particular, we are investigating extremely rare de novo mutations not found in the general population to find potential disease-associated rare variants^56)^. We also investigated postzygotic de novo mutations (mosaic or somatic mutations) in addition to classical de novo mutations (i.e., germline de novo mutations) to explore the unknown genetic architecture of bipolar disorder (Figure 2). While several studies have reported on mosaic mutations in ASD^57-60)^, pathological roles of mosaic mutations for psychiatric disorders remained known. Participants in our study were recruited through Bipolar Disorder Research Network Japan (BDRNJ, http://bipolar.umin.jp/), a network of patients, families, and researchers across Japan. Thanks to BDRNJ, we could report de novo mutation analysis with 354 families with bipolar disorder^56)^. This number is the world’s largest number for de novo mutation analysis in bipolar disorder to date. Research networks consisting of the patients and researchers such as BDRNJ are influential players in current genomic research.

Among the extremely rare de novo mutations in bipolar disorder, protein-truncating (loss-of-function) de novo mutation in a high probability of loss-of-function intolerance (pLI) genes is significantly more observed in bipolar disorder than in control. High pLI genes are the genes in which loss-of-function mutation is less observed in the general population than the theoretical estimation, meaning its susceptibility to natural selection by loss-of-function. This result is naturally expected for severe psychiatric disorders and is a common observation with schizophrenia and ASD^24, 40, 61)^. Among the extremely rare de novo mutations, deleterious mutations are enriched in the genes related to synapse and calcium ions. This result is consistent with the results of common variants and other biological studies on bipolar disorder^42, 62, 63)^, supporting the dysregulation of synapse and calcium signaling as the promising candidate mechanisms of bipolar disorder.

Among the genes hit by deleterious mutations found in this study, *KMT2C* is known to cause a severe neurodevelopmental disorder (Kleefstra syndrome^64)^) much more severe than bipolar disorder. We investigated the detail of the mutation in *KMT2C* to explain the apparent phenotypic difference between bipolar disorder and Kleefstra syndrome. Surprisingly, the mutation is, in fact, a mosaic mutation (postzygotic de novo mutation) in the proband’s body. The mutation exists only in some cells and should have occurred in the process of early development. Encouraged by this finding, we investigated postzygotic de novo mutations in patients with bipolar disorder. Among the postzygotic de novo mutations in bipolar disorder, deleterious mutations are enriched in the genes known to cause neurodevelopmental disorders (e.g., *KMT2C*). In addition, we found two deleterious mutations in *SRCAP* in two independent patients with bipolar disorder. *SRCAP* is known to cause a severe neurodevelopmental disorder, Floating-Harbor syndrome^65)^. This result leads to an interesting hypothesis: mosaic mutations in neurodevelopmental disorder genes cause milder phenotypes, including bipolar disorder. If this hypothesis holds true, bipolar disorder shares pathological mechanisms with severe neurodevelopmental disorders. Encouraged by rare variant studies on developmental disorders to understand the pathological mechanisms of psychiatric disorders^66)^, we are now proceeding with mosaic mutation analysis to confirm this hypothesis^67)^.

We conducted a comprehensive study of two types of mutations, germline and postzygotic (mosaic) de novo mutations, and elucidated a part of the genetic architecture of bipolar disorder^56)^. However, analysis with more families and patients with bipolar disorder is necessary to obtain more reliable and more profound knowledge. Future genomic research will be more empowered by research networks consisting of the patients and researchers such as BDRNJ. Through such collaboration, we will understand the pathological mechanisms and develop a new therapeutic/preventive strategy for bipolar disorder.

## Future direction: genotype first approach in psychiatry

In this Review, I have overviewed the current understanding of major psychiatric disorders by genomic investigation. Genomic analysis is one of the most powerful approaches to elucidate the biological mechanisms of psychiatric disorders. However, accumulated data from genomic studies so far proposes a critical question to the current diagnostic boundary among psychiatric disorders. Some genes are simultaneously associated with different disorders through common and rare variants^47-49)^. This is particularly notable for common variants associated with bipolar disorder and schizophrenia. In addition to the shared risk of common variants, *AKAP11*, the first gene reported to be associated with bipolar disorder through a gigantic meta-analysis of exome sequencing for rare variants, is also associated with schizophrenia^68)^. These results suggest that major psychiatric disorders share common pathological mechanisms, and prompt us to rethink the current disease concepts in psychiatry.

**Figure 3:**
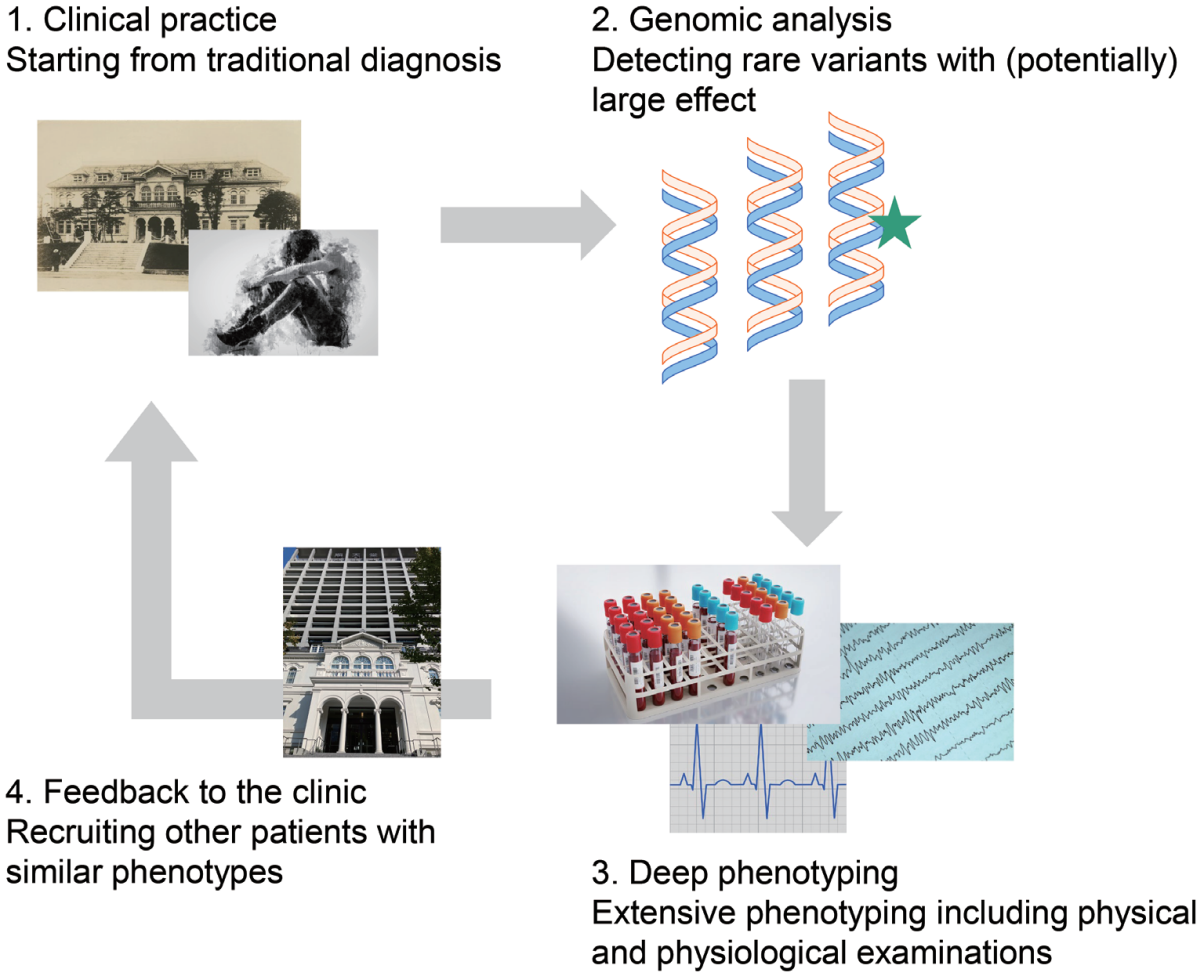
A schematic illustration of the genotype-first approach in our department (Department of Psychiatry, Faculty of Medicine, Juntendo University). The copyright-free images of clinical tests are derived from Pixabay (https://pixabay.com/). The historical image of the Juntendo clinic is derived with permission from Gakko-hojin Juntendo.

Researchers have started a new approach, the “genotype-first approach,” to resolve this issue ^69-73)^. The genotype-first approach is a research strategy to associate a specific genotype to a broad range of clinical phenotypes, not limited to conventional psychiatric symptoms but including somatic and physiological signs^74, 75)^. Traditional genetic research has adopted the opposite approach, the “phenotype-first approach,” which is a research strategy to associate a specific phenotype to genotypes. The genotype-first approach is essentially free from the current nosology of psychiatric disorders. This approach has begun to characterize the individuals with the established risk variants significantly affecting the onset of psychiatric disorders^76-80)^. Starting from the traditional diagnosis and genomic analysis, the researchers characterize the individuals with extensive medical evaluations including physical and physiological examinations to explore the genotype-phenotype correlation (Figure 3). The question is how much the genotype-first approach effectively characterizes possible disease concepts in a wide range of psychiatric disorders. Some clinicians have begun to feedback genetic information of such risk variants to the patients regardless of conventional psychiatric diagnosis^81)^. This approach will benefit future psychiatric research, but the answer will be evident through our effort with this genotype-first approach.

## Funding

This work was partly supported by AMED under Grant Number JP20dm0307028 (Strategic International Brain Science Research Promotion Program [Brain/MINDS Beyond] for M.N.), and JSPS KAKENHI under Grant Number JP18K15479 (M.N.), JP16H06277 (M.N.).

## Author Contributions

M.N. wrote and checked the manuscript.

## Conflict of interest statements

M.N. belongs to the Department of Molecular Pathology of Mood Disorders, Faculty of Medicine, Juntendo University, a joint laboratory of Juntendo University and Sumitomo Dainippon Pharma.
